# Brief guide to *Drosophila* maintenance and husbandry

**DOI:** 10.1016/j.mocell.2026.100349

**Published:** 2026-03-16

**Authors:** Chan Hoon Jung, Hyeonseong Kang, Yusung Park, Eunji Ahn, Jiwon Shim

**Affiliations:** School of Biological Sciences, Seoul National University, Gwanak-gu, Seoul 08826, Republic of Korea

**Keywords:** Balancer chromosome, *Drosophila melanogaster*, Fly husbandry, GAL4/UAS system, Genetic crossing

## Abstract

*Drosophila melanogaster* is a powerful genetic model organism due to its short life cycle, simple husbandry, and versatile genetic toolkit. Reliable experimental outcomes, however, depend on standardized husbandry and stock maintenance practices that are often underemphasized in research protocols. Here, we provide a concise, practical guide covering essential procedures for *Drosophila* culture conditions, anesthetization, phenotypic sorting, genetic crosses, developmental synchronization, and use of stock center resources. By documenting the standard methodologies and practices, this guide aims to help researchers, particularly trainees and newcomers, establish and maintain healthy fly stocks and minimize common pitfalls. These standardized practices will improve reproducibility and enable more effective use of the extensive genetic resources available in *Drosophila* research, thereby enhancing its utility across diverse fields of biology.

## Graphical Abstract

**Figure:**
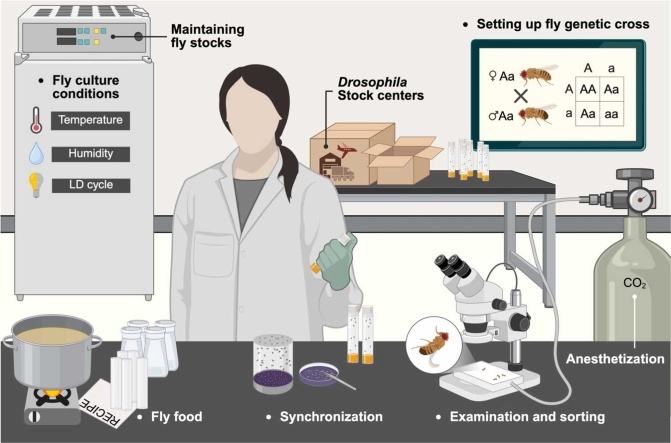

## BACKGROUND

Since Thomas H. Morgan established the fruit fly *Drosophila melanogaster* as a genetic model organism, *Drosophila* has become one of the most widely used systems in biological research due to its short life cycle, simple husbandry, and low maintenance cost. Its compact genome, organized into 4 discrete chromosomes, has greatly facilitated the discovery of general principles of heredity. Historically, several seminal advances propelled the field into modern *Drosophila* genetics: identification of the white-eyed mutant (Morgan, 1910), along with subsequent visible and heritable mutations generated by X-ray mutagenesis (Muller, 1927), accelerated understanding of complex relationships between genotype and phenotype and helped scientists in the modern era of genetics and molecular biology. Balancer chromosomes, special chromosomes containing multiple inversions that suppress recombination and carrying visible markers such as *Stubble* (*Sb*), *Curly* (*Cy*), *Tubby* (*Tb*), and *white* (*w*), enable fly geneticists to maintain mutant lines stably over long periods (Greenspan, 2004). The successful introduction of the GAL4/UAS system (Brand and Perrimon, 1993), a bipartite platform for spatially and temporally controlled gene expression *in vivo*, led to extensive collections of GAL4 driver and UAS responder lines that allow tissue-specific gene manipulation. In addition, genome-wide RNA interference libraries (Dietzl et al., 2007, Kennerdell and Carthew, 2000, Ni et al., 2008), which enable systematic loss-of-function screening by suppressing mRNA expression, have further expanded the *Drosophila* genetic toolkit. Together, these large collections of transgenic fly lines provide creative and fancy resources for investigating a wide range of biological questions, and these genetic resources are distributed worldwide through international *Drosophila* stock centers. In this guide, we provide essential information on practical procedures for maintaining *Drosophila* stocks, introduce major stock centers, and outline key aspects of husbandry to help beginners ensure reliable experimental use of the fruit fly model system.

### Fly husbandry

#### Fly culture conditions

**Fig. 1:**
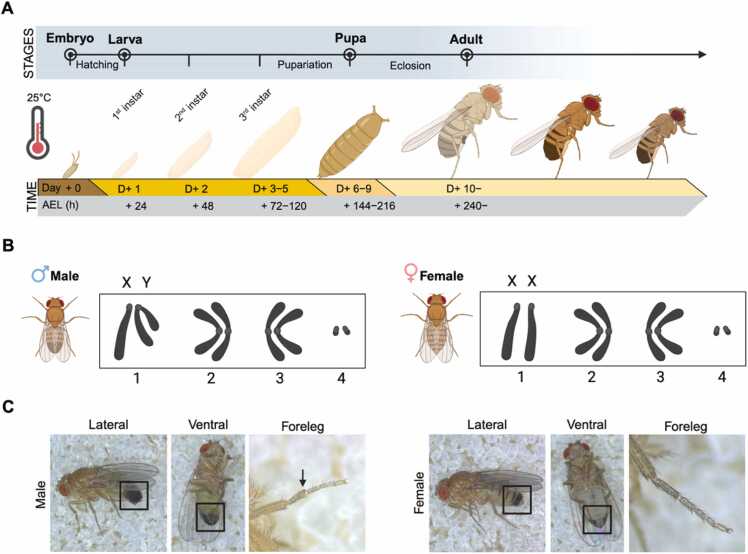
*Drosophila melanogaster* as a genetic model organism. (A) Life cycle of *Drosophila* at 25°C. Representative illustrations of *Drosophila* are shown sequentially. The top row indicates developmental stage, and the bottom row shows the time scale in days or hours after egg laying (AEL). (B) Karyotypes of *Drosophila* male and female. All *Drosophila* possess autosomal chromosome pairs 2, 3, and 4; males are XY, whereas females are XX. (C) Morphological features of *Drosophila* male and female. The lateral and Ventral views of the genital regions are highlighted with white squares, and the sex comb on the male foreleg is indicated by a white arrow.

*Drosophila melanogaster* originated in sub-Saharan Africa and has since spread worldwide (Chen et al., 2024). In nature, fruit flies are typically found near fermenting fruits or decaying plants, which serve as both feeding and oviposition sites. In the earliest *Drosophila* laboratories, glass milk bottles were used for culturing flies (Bridges, 1916, Morgan, 1910), but the field has evolved to use lightweight, transparent, and durable plastic containers of standardized sizes, most commonly cylindrical vials (ϕ 25×95 mm) or narrow-mouth bottles (56×56×100 mm). Cylindrical vials are well suited for small-sized cultures with approximately 20 to 30 adult flies and are commonly used for stock maintenance and genetic crosses, whereas bottles can accommodate 100 to 150 flies without overcrowding. Cotton or mesh plugs are used as lids, as they allow easy opening and closing while preventing flies from escaping, permitting gas exchange, and minimizing contamination. The most critical physical parameters for fly husbandry are temperature and humidity. Temperature, in particular, significantly affects developmental rate. For example, development from fertilized egg to adult takes approximately 19 days at 18°C, 10 days at 25°C, and 7 days at 29°C (Fig. 1A). Therefore, culture temperature can be adjusted to match experimental objectives. Laboratory stocks are often maintained at 18°C-25°C. Culture temperature can be manipulated up to 29°C to either activate or suppress GAL4-UAS-mediated gene expression (Mondal et al., 2007). Humidity is generally maintained at 50%-70% to prevent food desiccation at low humidity and to avoid mold growth or larval drowning at high humidity. In addition to these factors, a consistent light-dark cycle should be also considered to faithfully mimic the natural environment. As the diurnal activity of *Drosophila* is regulated by the fine interplay between environmental light and the internal circadian clock (Bargiello et al., 1984, Konopka and Benzer, 1971, Zehring et al., 1984), it is a particularly important factor for neurobiological research.

#### Fly food

Fruit flies are primarily saprophagous, feeding on rotten fruit and decaying plant material colonized by yeast and bacteria (Reaume and Sokolowski, 2006). In the laboratory, the earliest chemically defined media contained gelatin, dextrose, cholesterol, nucleic acids, and vitamins (Begg and Robertson, 1950, Sorge et al., 2025). Over time, convenient standardized diets were developed in laboratories. Standard *Drosophila* medium typically contains cornmeal, yeast, sugar, agar, and preservatives, which enhances experimental reproducibility and facilitates resource exchange between laboratories. Cornmeal serves as the principal macronutrient source, while additional grains such as malt extract, molasses, and soy flour can be included to improve dietary balance. Yeasts provide essential nutrients, including proteins, vitamins, and minerals, critical for larval development, creating a symbiotic relationship. Sugar, such as glucose, serves as a major energy source, and agar provides moisture and structural base. Although the basic composition of fly medium is standardized, exact ingredient ratios vary depending on the culture environment and experimental purposes. Each stock center often uses site-specific recipes that reflect different local climate, tradition, and available resources (Table 1). In general, a large batch of medium is prepared at once, dispensed into vials or bottles, and capped with cotton plugs. These can be stored for up to one month at 10-14°C.

**Table 1:** Fly food recipes used by *Drosophila* stock centers

		BDSC	VDRC	NIG-FLY	Kyoto-Fly	KDRC
Solid ingredients (g/L)	Agar	5.29	7.61	5.98	6.67	8.80
	(Dried) Yeast	15.88	17.10	79.72	37.07	42.30
	Cornmeal	67.09	76.07	39.86	64.88^a^	66.40
	Soy flour	9.18	9.51	-	-	-
	Malt extract	-	76.07	-	-	-
	Molasses	-	20.91	-	-	29.20
	Glucose (Dextrose)	-	-	99.66	-	-
Liquid ingredients (mL/L)	Corn syrup	70.62	-	-	-	-
	Propionic acid (>99%)	4.42	8.08	0.48	4.63	5.20
	Phosphoric acid	-	0.48	-	-	-
	Nipagin (15%)	-	11.40	-	-	-
	10% Methylparaben	-	-	2.99	6.94	10.50

Five major stock centers provide distinct standard food recipes on their websites (see Table 2). Prepare the food by dissolving agar powder in hot water, then adding the remaining solid ingredients and corn syrup. Add and mix preservatives at temperatures below 60°C before dispensing into vials or bottles.

a Corn flour:corn grits = 2:1.

#### Anesthetization

For precise genetic works using multiple transgenic or mutant stocks, individual fly in each stock must be carefully examined, sorted, and crossed under controlled conditions. Handling flies outside their housing always carries the risk of escapes, which can disrupt stock maintenance or compromise experiments. To minimize this risk and ensure proper manipulation, several methods have been developed to temporarily anesthetize flies. A range of fly anesthetics with different physicochemical properties, including ether vapor, cold, sevoflurane, eugenol, and triethylamine (Weineck et al., 2019), provides researchers options suitable for their specific purposes. Cold anesthesia, in which flies are exposed to 0-4°C by placing vials on ice or in a refrigerator, is the simplest method for reversibly immobilizing flies. Using carbon dioxide (CO_2_) is the most used and efficient method to anesthetize flies in laboratories. CO_2_ is a colorless, odorless, and stable gas that serves as an effective anesthetic by transiently inhibiting synaptic transmission at the neuromuscular junction (Badre et al., 2005). Flies exposed to a concentrated CO_2_ stream become anesthetized within a few seconds and gradually recover once the gas is removed. A CO_2_ pad with a meshwork provides a stable CO_2_ gas flow and a convenient workplace for examination and selection under a light microscope. To anesthetize flies in vials or bottles, simply flip them on the CO_2_ pad after removing a cap. Alternatively, CO_2_ gas can be introduced into the containers using a needle-like gas sprayer before entirely opening the cap. When using the later approach, researchers tilt the container to prevent immobilized flies from drowning into the food. The installation and operation of CO_2_ supply system should comply with institutional safety guidelines: use regulators and/or valves to monitor and control flow pressure and store gas tanks in a secure, designated location. Optimal flow settings should be determined empirically in each laboratory because they depend on equipment and environmental conditions. Note that prolonged CO_2_ exposure, over 10 min, can adversely affect fly health and fertility and may compromise subsequent experiments (Zimmerman and Berg, 2024). Therefore, it is important to minimize exposure time whenever possible.

#### Examination and sorting

Central to fly genetics is the principle that distinct visible phenotypes correspond to specific underlying genotypes, allowing researchers to infer genotypes from external morphology. Commonly used external markers include eye color, eye shape, wing shape, and bristle length, all of which are readily distinguished using a stereomicroscopy. Morphological examination is usually conducted on a CO_2_ pad, where immobilized flies are selected and transferred to vials using a soft paintbrush (sizes from 0 to 5). In addition, sex is an intuitive and essential trait determined by the sex chromosomes (Fig. 1B) (Erickson and Quintero, 2007). Females are larger, especially in the abdomen, and have pale pointed genitalia, whereas males display darker and rounded genital claspers and sex combs on the forelegs (Fig. 1C). With experience, fly researchers learn to sort individuals by phenotype and sex to assemble the desired genotypes. If a fly escapes during sorting, it should be captured and discarded rather than returned to the stock, since escaped flies can introduce pathogens or unwanted genetic backgrounds and thus compromise experiments.

#### Setting up fly genetic cross

**Fig. 2:**
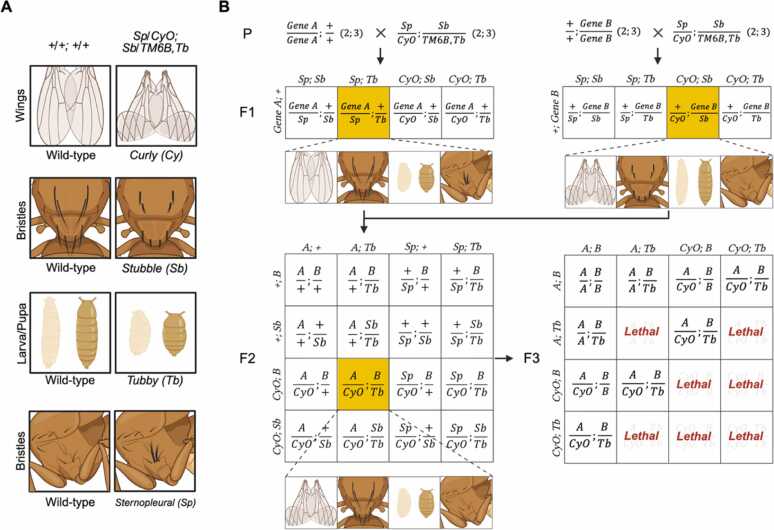
*Drosophila* balancer strains and an example of cross scheme. (A) A balancer strain (*Sp/CyO*; *Sb/TM6B, Tb* (2; 3)) carries dominant visible markers that distinguish it from wild-type flies (*+/+*; *+/+* (2; 3)). Curly wings indicate the presence of the second chromosome balancer, *CyO*, and the tubby (*Tb*) short and squat larvae-pupal body phenotype indicates the third chromosome balancer, *TM6B*. Additional visible markers, such as stubble bristles on the dorsal thorax (*Sb*) and sternopleural (*Sp*) bristles on the lateral thorax, are used to follow balancer chromosomes in crosses. (B) An example cross scheme to combine 2 genes of interest, *Gene A* (chromosome 2) and *Gene B* (chromosome 3). Each homozygous parent is crossed with the balancer strain (P generation). F1 progeny with the desired genotype (*Gene A*/*Sp*; +/*TM6B, Tb*, and +/*CyO*; *Gene B*/*Sb*), indicated in yellow squares, are selected and intercrossed. The resulting F2 progeny carrying the genotype (*Gene A/CyO*; *Gene B/TM6B, Tb*) are sorted based on their visible markers. This genotype can be stably maintained. F3 offspring from this stock inherit both *Gene A* and *B*. Animals homozygous for balancer chromosomes are lethal.

With a clear understanding of genetic mechanisms underlying fly heredity, geneticists can predict progeny genotypes from parents with known genotypes by calculating the possible outcomes. This predictive framework enables the design of genetic crosses that yield the desired genotypes. A practical genetic tool for generating and maintaining specific fly genotypes is the balancer chromosome (Fig. 2B). Balancer chromosomes facilitate genotype tracking through dominant visible marker phenotypes and prevent the loss of alleles by suppressing recombination (Fig. 2A) (Greenspan, 2004, Kaufman, 2017). After planning a cross, selected males and females are placed together in a vial, typically at a 1:1 ratio, although this may be adjusted depending on the genotypes. In general, the number of females can be slightly increased relative to males to ensure effective mating. Mating vials are flipped daily or every other day to fresh food vials to avoid overcrowding and maintain controlled experimental conditions. For genetic crosses, it is necessary to use virgin females that have never mated. Once mated, females can store sperm in the seminal receptacle and spermathecae and use it to fertilize eggs for up to 2 weeks, which makes it difficult to track the genotypes of fertilized eggs (Fowler, 1973, Manier et al., 2010). Collecting virgin females is therefore the first step in most genetic crosses. Since it takes approximately 8 hours for flies to become sexually mature after eclosion, newly eclosed females are typically virgin during this 8-hour time window. Virgin females often have pale, soft cuticles due to immature pigmentation, a relatively large abdomen, unfolded wings, and a distinct dark spot on the lateral abdomen corresponding to meconium-like waste material accumulated during the pupal stage. As *Drosophila* predominantly ecloses at dawn or in the early morning under a standard 12:12 light-dark cycle (Myers et al., 2003) that maintains a 24-hour circadian rhythm, collecting virgins every morning is an efficient strategy for setting up fly crosses.

#### Synchronization

**Fig. 3:**
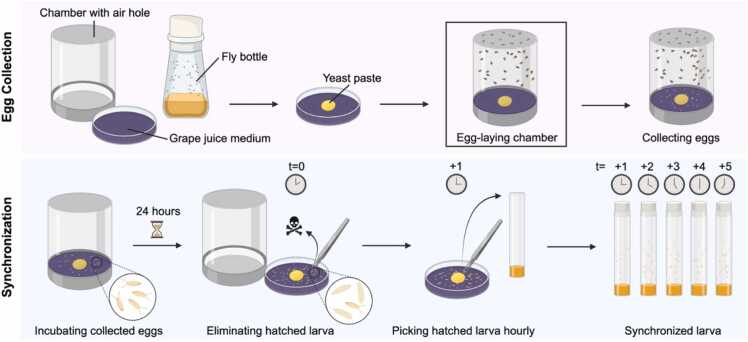
Developmental synchronization of *Drosophila* larvae. Yeast paste, which promote *Drosophila* oviposition, is placed on the center of grape juice medium. A clear plastic chamber with air hole covers the medium to prevent flies from escaping. Eggs are collected in the egg-laying chamber for a defined period of time. After the flies are completely removed, the eggs are incubated for 24 h. Already hatched larva is removed with forceps (*t* = 0) and newly hatched larvae are collected into fresh vials every hour (*t*=+n). Each vial contains developmentally synchronized larvae.

Since *Drosophila* has a rapid life cycle, its behavior and physiology change markedly across developmental stages (Arbeitman et al., 2002). Consequently, visible and measurable phenotypes led by specific genetic mutations may be transient or vary with developmental stages (Shin et al., 2000). Female flies lay fertilized eggs daily for up to 3 weeks, so it is common to find flies at multiple stages, from first instar larvae to pupae and adults, within a single vial or bottle. Mixed stages complicate phenotypic analysis of genetic backgrounds of interest. Thus, synchronizing the developmental stage of progeny is necessary for reproducible and reliable experiments. To synchronize developmental stages, a large number of adult flies are placed into an egg-collection chamber, consisting of a plastic cage fitted with a yeast-pasted grape juice agar plate, which promotes oviposition and provides food for hatched larvae (Becher et al., 2012). After a defined egg-lay period, for example, 6 hours, researchers wait another 24 hours and remove all hatched first-instar larvae. Newly hatched first-instar larvae are then transferred to individual fresh vials at hourly intervals so that each cohort collected is developmentally synchronized within a 1-hour time window (Fig. 3). As development proceeds on a predictable timeline at a constant temperature, this approach enables accurate scheduling of subsequent experiments with minimal developmental variability.

### Ordering fly stocks

#### Drosophila stock centers

There are over 2,000 laboratories working with flies worldwide, and research using the fruit fly has expanded remarkably, producing more than 1,000 publications annually in this century (Ayala et al., 2010). Central to this progress are international *Drosophila* stock centers, which curate and maintain massive collections of strains for the global research community (Cook and Parks, 2022). These centers also receive stock donations from individual labs, distributes strains on demand, and generate new fly lines that incorporate emerging technologies. The Bloomington Drosophila Stock Center, originating from the roots of fly biology, has grown into the world’s largest stock bank and currently maintains roughly 90,000 strains. Other major repositories include the Vienna Drosophila Resource Center in Austria, the FlyORF in Switzerland, the National Institute of Genetics-Fly Division (NIG-FLY) and the Kyoto Drosophila Stock Center (Kyoto-Fly) in Japan, the Korean Drosophila Resource Center in Korea, and the Tsinghua Fly Center in China; each provides unique stock collections that reflect its missions. The National Drosophila Species Stock Center distributes non-*melanogaster* species (Table 2). All stock centers publish their entire catalogs and husbandry information online, which allow researchers to create accounts, add stocks to a cart, and order stocks for delivery to their labs. Fly stocks are typically shipped as duplicate plastic vials containing standard medium to minimize contamination and ensure survival during shipment.

**Table 2:** A list of *Drosophila* stock centers

Center	Location (URL)	Major resources	No. of stocks	Other services
BDSC	Indiana University Bloomington, USA (https://bdsc.indiana.edu/)	Balancers, GAL4/UAS, GAL80, RNAi, CRISPR	>90,000	Fly care information Stock donation
VDRC	Vienna Biocenter Core Facilities GmbH, Austria (https://shop.vbc.ac.at/vdrc_store/)	RNAi, CRISPR	>37,000	Private stock keeping On-site screening Stock donation
NIG-FLY	National Institute of Genetics Mishima, Japan (https://shigen.nig.ac.jp/fly/nigfly/)	RNAi, KO, CRISPR	>18,000	Segmentation antibodies Cas9 reagents Stock donation
Kyoto-Fly	Kyoto Institute of Technology, Japan (https://kyotofly.kit.jp/)	Balancers, RNAi, GAL4/UAS	>25,000	Stock donation
KDRC	Gwangju Institute of Science and Technology, Republic of Korea (http://kdrc.kr/)	Balancers, GAL4/UAS, GenExel EP	>7,800	Plasmid microinjection Fly gut image database Stock donation
THFC	Tsinghua University Beijing, China (https://thfc.zzbd.org/en/)	RNAi, CRISPR, GAL4/UAS	>8,000	Microinjection CRISPR mutant generation Stock donation
NDSSC	Cornell College of Agriculture and Life Science, New York, USA (https://www.drosophilaspecies.com/)	*Drosophila*, *Sophophora*	>250	Food recipes for specialist groups of Drosophilidae
Species stock exchange	*Drosophila* species stock exchange (email to: *br.hopkins@ufl.edu*)	*Drosophila, Chymomyza*	>100	Collecting and distributing *Drosophila* species

Seven major stock centers worldwide provide detailed information about their unique collections and available services on their websites. Comprehensive databases such as FlyBase (https://flybase.org/) or the Drosophila Genetic Resource Center (https://dgrc.bio.indiana.edu/) serve as online platforms that connect researchers to these stock centers.

#### Maintaining fly stocks

Each vial in the laboratory stock collection should be clearly labeled with relevant information, including the genotype with chromosome numbers, stock number, culture start date, and the presence of a backup vial. The flipping interval depends on the culture temperature: 1-2 weeks at 29°C, 2-3 weeks at 25°C, and 6-7 weeks at 18°C to maintain healthy stocks. Used vials and biological waste should be autoclaved or frozen at –20°C for 1-2 days to ensure all flies are completely killed before disposal, thereby minimizing the risk of releasing genetically modified animals. Central to stock maintenance is the prevention and treatment of contamination. Contamination by pests, including mites, fungi, bacteria, and viruses, poses a major threat to stock integrity (Park et al., 2005). Mite infestation is often observed on flies or on the food surface under light microscopy and may even visible to the naked eye. The most common risk factor for mite infestation is the introduction of newly arrived stocks from other facilities. These stocks should be quarantined before being integrated into a laboratory stock collection. In the quarantine area, newly arrived flies are transferred to fresh vials, and the original vials are retained as a backup. As they reproduce, the progeny of each generation are transferred to new vials and examined for mites. After 2 or more generations, progeny free of mite contamination can be incorporated into healthy stocks. If mites are detected, the contaminated flies are cultured in the presence of a miticide and flipped to fresh vials every 2 days until the mites are completely eliminated. Regular preventive measures, such as using appropriate preservatives or antibiotics in the food, sanitizing workspace and tools before handling stock vials, and maintaining regular flipping schedule, are also required to prevent common sources of contamination. The Bloomington *Drosophila* stock center provides detailed practical information on fly culture (https://bdsc.indiana.edu/information/fly-culture.html), and brief explanations and solutions to common problems in stock maintenance are summarized in Table 3.

**Table 3:** Potential issues in *Drosophila* stock management

Problem	Possible reason	Solution
Dead on arrival (DOA)	Extreme weather, long delivery period	Take a picture of the DOA vials and submit a reshipment request as soon as possible, in compliance with the policies of the respective stock center
No oviposition	Aged parental flies, extreme humidity or temperature	Set up crosses with newly eclosed individuals less than 10 days old, check temperature and humidity, add yeast paste
No or delayed pupation	Dry or wet food, poor food quality, overcrowding, infection, low or high temperature	Set up a new cross in fresh vials and keep them under disinfected conditions
Fungal contamination in fly stocks	Airborne spores landing on, high humidity and low temperature	Disinfect fungal spore in the air by using a HEPA air filter and maintain relative humidity below 60%, flip regularly
Dry fly food	Not being properly sealed	Keep fly food in a sealed bag to preserve humidity, once opened, use without delay
Changes in the life cycle period	Changes in temperature or light-dark cycle	Check light-dark cycle and temperature setting

Six common problems and their corresponding solutions are summarized. Issues in stock maintenance are mainly attributable to the condition of the fly food and environmental factors such as temperature and humidity.

## CONCLUDING REMARKS

Here, we introduce fundamental guidelines for *Drosophila* husbandry and stock maintenance, providing concise methodological guidance to support understanding of core *Drosophila* genetics workflows. Proper stock maintenance and careful handling of individual flies are essential for obtaining reliable experimental results. Although specific procedures and conditions may vary with laboratory environment and *Drosophila* species and strains, this guide summarizes common methods and core principles that serves as a practical foundation for initiating and sustaining fly research.

## Funding and support

This work was supported by Basic Science Research Program through the National Research Foundation of Korea (NRF) funded by the Ministry of Education (RS-2024-00349703 and RS-2025-02232977) and the New Faculty Startup Fund from Seoul National University to J.S.

## Author Contributions

CHJ, HK, YP, EA, and JS wrote and edited the manuscript. JS supervised the work and secured the fundings.

## Declaration of Competing Interests

The authors declare no competing interests.

## References

[bib1] Arbeitman M.N., Furlong E.E.M., Imam F., Johnson E., Null B.H., Baker B.S., Krasnow M.A., Scott M.P., Davis R.W., White K.P. (2002). Gene expression during the life cycle of *Drosophila melanogaster*. Science.

[bib2] Ayala F., Michán L., Castañeda Sortibrán A., Rodríguez-Arnaiz R. (2010). Global drosophila research: a bibliometric analysis. Drosoph. Inf. Serv..

[bib3] Badre N.H., Martin M.E., Cooper R.L. (2005). The physiological and behavioral effects of carbon dioxide on *Drosophila melanogaster* larvae. Comp. Biochem. Physiol. A Mol. Integr. Physiol..

[bib4] Bargiello T.A., Jackson F.R., Young M.W. (1984). Restoration of circadian behavioural rhythms by gene transfer in Drosophila. Nature.

[bib5] Becher P.G., Flick G., Rozpędowska E., Schmidt A., Hagman A., Lebreton S., Larsson M.C., Hansson B.S., Piškur J., Witzgall P. (2012). Yeast, not fruit volatiles mediate *Drosophila melanogaster* attraction, oviposition and development. Funct. Ecol..

[bib6] Begg M., Robertson F.W. (1950). The nutritional requirements of *Drosophila melanogaster*. J. Exp. Biol..

[bib7] Brand A.H., Perrimon N. (1993). Targeted gene expression as a means of altering cell fates and generating dominant phenotypes. Development.

[bib8] Bridges C.B. (1916). Non-disjunction as proof of the chromosome theory of heredity. Genetics.

[bib9] Chen J., Liu C., Li W., Zhang W., Wang Y., Clark A.G., Lu J. (2024). From sub-Saharan Africa to China: evolutionary history and adaptation of *Drosophila melanogaster* revealed by population genomics. Sci. Adv..

[bib10] Cook K.R., Parks A.L. (2022). The international exchange of *Drosophila melanogaster* strains. Rev. Sci. Tech..

[bib11] Dietzl G., Chen D., Schnorrer F., Su K.-C., Barinova Y., Fellner M., Gasser B., Kinsey K., Oppel S., Scheiblauer S. (2007). A genome-wide transgenic RNAi library for conditional gene inactivation in Drosophila. Nature.

[bib12] Erickson J.W., Quintero J.J. (2007). Indirect effects of ploidy suggest X chromosome dose, not the X:A ratio, signals sex in Drosophila. PLoS Biol..

[bib13] Fowler G.L., Caspari E.W. (1973).

[bib14] Greenspan R.J. (2004). Fly Pushing: The Theory and Practice of Drosophila Genetics.

[bib15] Kaufman T.C. (2017). A short history and description of *Drosophila melanogaster* classical genetics: chromosome aberrations, forward genetic screens, and the nature of mutations. Genetics.

[bib16] Kennerdell J.R., Carthew R.W. (2000). Heritable gene silencing in Drosophila using double-stranded RNA. Nat. Biotechnol..

[bib17] Konopka R.J., Benzer S. (1971). Clock mutants of *Drosophila melanogaster*. Proc. Natl. Acad. Sci. U. S. A..

[bib18] Manier M.K., Belote J.M., Berben K.S., Novikov D., Stuart W.T., Pitnick S. (2010). Resolving mechanisms of competitive fertilization success in *Drosophila melanogaster*. Science.

[bib19] Mondal K., Dastidar A.G., Singh G., Madhusudhanan S., Gande S.L., VijayRaghavan K., Varadarajan R. (2007). Design and isolation of temperature-sensitive mutants of Gal4 in yeast and drosophila. J. Mol. Biol..

[bib20] Morgan T.H. (1910). Sex limited inheritance in *Drosophila*. Science.

[bib21] Muller H.J. (1927). Artificial transmutation of the gene. Science.

[bib22] Myers E.M., Yu J., Sehgal A. (2003). Circadian control of eclosion: interaction between a central and peripheral clock in *Drosophila melanogaster*. Curr. Biol..

[bib23] Ni J.Q., Markstein M., Binari R., Pfeiffer B., Liu L.P., Villalta C., Booker M., Perkins L., Perrimon N. (2008). Vector and parameters for targeted transgenic RNA interference in *Drosophila melanogaster*. Nat. Methods.

[bib24] Park S.-Y., Heo Y.-J., Kim K.-S., Cho Y.-H. (2005). *Drosophila melanogaster* is susceptible to vibrio cholerae infection. Mol. Cells.

[bib25] Reaume C.J., Sokolowski M.B. (2006). The nature of *Drosophila melanogaster*. Curr. Biol..

[bib26] Shin E.C., Cho S.E., Lee D.-K., Hur M.-W., Paik S.R., Park J.H., Kim J. (2000). Expression patterns of α-synuclein in human hematopoietic cells and in drosophila at different developmental stages. Mol. Cells.

[bib27] Sorge S., Girard V., Lampe L., Tixier V., Weaver A., Higgins T., Gould A.P. (2025). A Drosophila holidic diet optimized for growth and development. Dev. Cell.

[bib28] Weineck K., Stanback A., Cooper R.L. (2019). The effects of eugenol as an anesthetic for an insect: Drosophila, adults, larval heart rate, and synaptic transmission. Proc. Assoc. Biol. Lab. Educ..

[bib29] Zehring W.A., Wheeler D.A., Reddy P., Konopka R.J., Kyriacou C.P., Rosbash M., Hall J.C. (1984). P-element transformation with period locus DNA restores rhythmicity to mutant, arrhythmic *Drosophila melanogaster*. Cell.

[bib30] Zimmerman S.G., Berg C.A. (2024). CO_2_ exposure drives a rapid pH response in live adult Drosophila. PLoS One.

